# Effect of Chemotherapeutics and Tocopherols on MCF-7 Breast Adenocarcinoma and KGN Ovarian Carcinoma Cell Lines* In Vitro*

**DOI:** 10.1155/2019/6146972

**Published:** 2019-01-15

**Authors:** Daniela Figueroa, Mohammad Asaduzzaman, Fiona Young

**Affiliations:** Department of Medical Biotechnology, College of Medicine and Public Health, Flinders University, Adelaide, SA, 5052, Australia

## Abstract

The combination of doxorubicin and cyclophosphamide commonly used to treat breast cancer can cause premature ovarian failure and infertility. *α*-Tocopherol is a potent antioxidant whereas *γ*-tocopherol causes apoptosis in a variety of cancer models* in vitro* including breast cancer. We hypothesised that the combination of doxorubicin (Dox) and 4-hydroperoxycyclophosphamide (4-Cyc) would be more cytotoxic* in vitro* than each agent alone, and that *α*-tocopherol would reduce and *γ*-tocopherol would augment the cytotoxicity of the combined chemotherapeutics. Human MCF-7 breast cancer and KGN ovarian cells were exposed to Dox, 4-Cyc, combined Dox and 4-Cyc, *α*-tocopherol, *γ*-tocopherol, or a combination of Dox and 4-Cyc with *α*-tocopherol or *γ*–tocopherol. Cell viability was assessed using a crystal violet assay according to four schedules: 24h exposure, 24h exposure + 24h culture in medium, 24h exposure + 48h culture in medium, or 72h continuous exposure. Supernatants from each separate KGN culture experiment (n=3) were examined using an estradiol ELISA. Dox was cytotoxic to both MCF-7 and KGN cells, but 4-Cyc only killed MCF-7 cells. *γ*-Tocopherol significantly decreased MCF-7 but not KGN cell viability. The combined chemotherapeutics and *γ*-tocopherol were more cytotoxic to MCF-7 than KGN cells, and *α*-tocopherol reduced the cytotoxicity of the combined chemotherapeutics towards KGN ovarian cells, but not MCF-7 cells. The addition of both *γ*-tocopherol and *α*-tocopherol to the chemotherapeutic combination of Dox and cyclophosphamide has the potential to increase* in vitro* chemotherapeutic efficacy against breast cancer cells whilst decreasing cytotoxicity towards ovarian granulosa cells.

## 1. Introduction

In Asia, approximately 25% of all breast cancer patients are premenopausal and younger than 35 years old [1]. Worldwide, up to 90% of breast cancer patients can survive for 5 years following diagnosis [2, 3] but it was found that chemotherapy-induced premature ovarian failure and infertility reduce the survivors quality of life [4–10].

Many types of breast cancer are treated with a combination of chemotherapeutic agents such as doxorubicin (adriamycin) and cyclophosphamide [3, 11, 12]. Clinical administration [13, 14] resulted in plasma concentrations of 1.8±0.4*μ*M doxorubicin within 24h of infusion [15] and serum concentrations of 4-hydroxycyclophosphamide to be approximately 0.02uM 2-4h after administration [16].

Cyclophosphamide, an alkylating agent, requires hepatic activation to form 4-hydroxycyclophosphamide and aldophosphamide, which coexist in equilibrium and diffuse freely into cells. Aldophosphamide is metabolised into phosphoramide mustard [17, 18] which causes intra- and interstrand crosslinking in DNA. This interferes with DNA replication [19] and stimulates apoptosis [17]. A synthetic compound, 4-hydroperoxycyclophosphamide (4-Cyc), is metabolised to 4-hydroxycyclophosphamide* in vitro* [13, 20] and* in vivo* [21, 22]. Aldehyde dehydrogenase oxidises aldophosphamide to an inactive metabolite instead of the active phosphoramide mustard, and hence cells with different levels of aldehyde dehydrogenase respond differently to 4-Cyc [18].

Doxorubicin (Dox), an anthracycline agent, intercalates at double strand DNA breaks in a topoisomerase-II dependent manner and inhibits DNA replication, synthesis, and mitosis [23, 24]. Dox also induces the production of reactive oxygen species (ROS) which cause lipid peroxidation and apoptosis [25]. The combined administration of both drugs caused therapeutic synergism in a mouse model [26] that was attributed to these different mechanisms of action: cyclophosphamide crosslinking of DNA strands and Dox prevention of DNA repair [27].

The chemotherapeutic combination of Dox and cyclophosphamide causes premature ovarian failure in premenopausal breast cancer patients [10, 18, 28]. Ovaries contain follicles, a spherical structure consisting of a single oocyte (egg) surrounded by layers of dividing granulosa cells. Granulosa cells produce anti-Müllerian hormone (AMH) which inhibits activation of small, quiescent primordial follicles [29]. It is thought that chemotherapeutics cause granulosa cell death [30, 31], which reduces AMH and results in the activation of primordial follicles [10]. The granulosa cells in the activated follicles proliferate and the follicles grow, but subsequent cycles of Dox and cyclophosphamide therapy cause granulosa cell death and loss of these follicles [32, 33]. Hence chemotherapy to treat breast cancer reduces serum concentrations of AMH, depletes the ovary of its reservoir of quiescent primordial follicles, and advances infertility through premature ovarian failure [10, 34]. The administration of cyclophosphamide to rodents caused a dose-dependent loss of small follicles [32, 35, 36] with DNA double strand breaks in the oocytes [37]. Dox caused apoptosis in mature murine oocytes [38, 39] and the* in vivo* administration of Dox to mice significantly reduced the numbers of follicles, whilst increasing ovarian apoptosis [40, 41]. It is clear that cyclophosphamide alone, or Dox alone, has adverse effects on the follicular granulosa cells of the ovary, but there are no reports describing the cytotoxic effects of the combined regime (which is used to treat breast cancer patients) on ovarian granulosa cells.

Dox-induced ROS damage was significantly lower in mice administered vitamin E [42, 43], and vitamin E decreased the toxicity of Dox without reducing its effectiveness as chemotherapeutic agent [44–49]. Vitamin E consists of eight structurally distinct compounds classified as tocopherols (alpha, beta, gamma, and delta) and tocotrienols (alpha, beta, gamma, and delta) [50–53]. Tocopherols have antioxidant activity against ROS-induced lipid peroxidation [54, 55], and gamma tocopherol (*γ*Toc) is the prominent form in the human diet [56].

The administration of *α*-tocopherol (*α*Toc) to 21 breast cancer patients prior to chemotherapy significantly elevated serum concentrations of *α*Toc but did not augment efficacy of the chemotherapeutics and did not decrease toxic side-effects, although ovarian function was not assessed in this study [57]. It seems that long-term dietary supplementation with antioxidant vitamins reduces the incidence, but not the severity, of cancer [58, 59]. Klein et al. [60] reported that *α*Toc did not have anticancer properties* in vivo*, but when the human breast cancer MCF-7 cell line was used to generate tumours in mice, the dietary administration of either *α*Toc or *γ*Toc reduced tumour growth [53]. Delta and *γ*Toc increased the levels of proapoptotic proteins, inhibited expression of antiapoptotic proteins* in vivo,* and also had antitumour activity in animal models of colon and prostate cancer [52]. *γ*Toc inhibited the proliferation of human breast cancer cells* in vitro* [52, 61], delayed the formation of breast cancer tumours in rodent models [52], and induced apoptosis in breast cancer cells via upregulation of DR5 expression [60]. Estrogen metabolism can generate ROS and this may contribute to the pathogenesis of breast cancer [53]. This also suggests that antioxidant tocopherols may have more anticancer activity* in vivo* than in estrogen-free* in vitro* systems.

We hypothesised that the combination of Dox and cyclophosphamide would be more cytotoxic* in vitro* to the human MCF-7 breast cancer cell line and the human ovarian granulosa tumour-derived KGN cell line than each chemotherapeutic agent alone [26]. Both alpha and gamma tocopherol are antioxidants with the potential to reduce chemotherapeutic-induced ROS damage and consequently reduce cytotoxicity, but *γ*Toc additionally has anticancer activity. We therefore hypothesised that *γ*Toc, but not *α*Toc, would augment the cytotoxic activity of the combined Dox and cyclophosphamide regime* in vitro.*

## 2. Materials and Methods

### 2.1. Chemicals and Reagents

All chemicals and reagents used in this study were obtained from Sigma-Aldrich (Australia), unless specified otherwise.

### 2.2. Preparation of Solutions

Stock solutions of 100*μ*M doxorubicin (Dox) and 1000*μ*M 4-hydroperoxycyclophosphamide (4-Cyc, ThermoFisher Scientific, Victoria, Australia) were prepared in RPMI media and 10% foetal calf serum (FCS, DKSH, Victoria, Australia) for MCF-7 cells or in DMEM/F12 media and 10% FCS for KGN cells. These solutions were kept at 4°C and -20°C, respectively, for a maximum of 3 months and were diluted immediately before use, because these conditions maintain activity and stability [62, 63]. Stock solutions of alpha and gamma tocopherol (*α*Toc and *γ*Toc) were prepared by diluting the compounds in dimethyl sulfoxide (DMSO) to yield solutions of 1000*μ*M. These were stored for a maximum of 3 months at 4°C. Further dilutions in the appropriate cell culture medium were prepared immediately before use, and cells were exposed to 0.8% DMSO. The 0.5% crystal violet stain was prepared in a 50% methanol (99.9% pure). 100% acetic acid was diluted to 33% with demineralised water, to be used as a destaining solution in the crystal violet assay.

### 2.3. Cell Culture

The MCF-7 human epithelial breast adenocarcinoma cell line was obtained from the America Type Culture Collection (ATCC) and maintained in RPMI media, supplemented with 10% FCS and 1% v/v of 10,000 units/mL penicillin + 10mg/mL streptomycin. Media were replaced every 2-3 days and cells were harvested with 0.1% trypsin/EDTA solution and subcultured twice a week. The KGN human granulosa carcinoma cell line [64] was kindly donated by Dr. Theresa Hickey, Research Centre for Reproductive Health, Department of Obstetrics and Gynaecology, University of Adelaide, and maintained in DMEM/F12 supplemented with insulin (5*μ*g/mL), transferrin (5*μ*g/mL), selenium (5ng/mL, ITS), 10% FCS, and 1% v/v of 10,000 units/mL penicillin + 10mg/mL streptomycin. Although the KGN cell line was derived from an ovarian granulosa cell carcinoma, it can be used as a model for human ovarian granulosa cell growth, apoptosis, and steroid hormone production [64]. Media were replaced every 2-3 days and both cell lines were subcultured twice a week. Cell culture flasks containing 80% confluent cells in exponential growth phase were used for all experiments.

### 2.4. Effect of Doxorubicin, 4-Hydroperoxycyclophosphamide, and *α*- and *γ*-Tocopherol on MCF-7 and KGN Cell Viability

MCF-7 cells (20,000 cells per well) and KGN cells (25,000 cells per well) were added to 96-well microplates. After a 24h adherence period, supernatants were removed and cells were exposed to 100*μ*L of chemotherapeutics or tocopherols (Table 1). The chemotherapeutic doses selected for this* in vitro* study bracket the clinical,* in vivo *serum concentrations of Dox [15] and 4-hydroxycyclophosphamide [16] (Table 1). Cells were exposed to chemotherapeutics and tocopherols according to four different schedules: 24h exposure, 24h exposure + 24h culture in media, 24h exposure + 48h culture in media, or 72h continuous exposure where reagents in medium + 10% FCS were replenished every 24h. After exposure to chemotherapeutics and tocopherols, media containing reagents were collected and frozen, and the cell viability was assessed by the crystal violet (CV) assay. Each test condition was examined in three replicate wells and each experiment was repeated on 3 separate occasions (n=3) for the two cell types.

### 2.5. Crystal Violet (CV) Cell Viability Assay

Cell culture supernatants were replaced with 50*μ*L of crystal violet stain (0.5%). The cells were stained and fixed for 10min at room temperature. Excess stain was rinsed away with demineralised water, and cells were left to air-dry overnight. 50*μ*L of destaining solution was added for 10min. The optical density was read at 570nm with correction at 630nm [65]. A crystal violet standard plot was produced in each replicate experiment in which MCF-7 cell densities ranged from 0 to 80,000 and KGN cell densities from 0 to 100,000 cells per well in replicates of 6 for each cell density. Absorbance readings were plotted against cell densities with an average linear correlation of R^2^ = 0.99 (n=3) replicate experiments for MCF-7 cells and R^2^ = 0.97 (n=3) replicate experiments for KGN cells. Numbers of viable cells after exposure to chemotherapeutics and/or tocopherols were determined by comparison with the CV standard curve for the same experimental replicate.

### 2.6. Estradiol Enzyme-Linked Immunosorbent Assay (ELISA)

Supernatants from each KGN culture experiment (n=3) were examined in a competitive estradiol ELISA (Cayman Chemical ELISA, Ann Arbor, MI, USA) that uses a mouse anti-rabbit IgG and an acetylcholinesterase estradiol tracer. Detection ranges from 6.6 to 4000 pg/mL, and the intra-assay coefficient of variation (CoV) ranges from 7.8 to 18.8%. For this study, the estradiol standard was diluted in the DMEM/F12 cell culture medium to give concentrations that ranged from 6.6 to 4000 pg/mL. A separate standard plot was constructed for each experimental replicate (n=3) and the lowest R^2^ value was 0.99. Concentration of estrogen was determined by comparison with the standard curve. Estrogen/cell concentration was calculated by dividing pg/mL of estrogen for each culture well by the numbers of viable cells in the same well.

## 3. Statistical Analysis

To examine the dose-dependent effect of chemotherapeutics and/or tocopherols, a one-way ANOVA with Tukey HSD and Bonferroni post hoc was conducted. To examine the effect of the four different exposure schedules on cell viability, an ANOVA was conducted that used the periods of culture as independent factors. Statistical significance was assessed by Tukey HSD and Bonferroni post hoc tests. A one-way ANOVA with Tukey HSD post hoc was conducted to examine estrogen production. These statistical analyses were performed using SPSS statistics software (V22.0 IBM, Australia). Statistical significance was set at p ≤ 0.05. All experiments were performed as three independent replicates, and all data expressed as mean ± standard deviation.

## 4. Results

KGN (25,000) and MCF-7 (20,000) cells were added to each well, and after 24h adherence and 24h culture in control conditions, there were 113,600±15,600 KGN cells/well and 38,100±4400 MCF-7 cells/well. After 24h adherence and 72h in culture there were 119072±8750 KGN and 83383±13546 MCF-7 cells per well in control medium.

**Figure 1:**
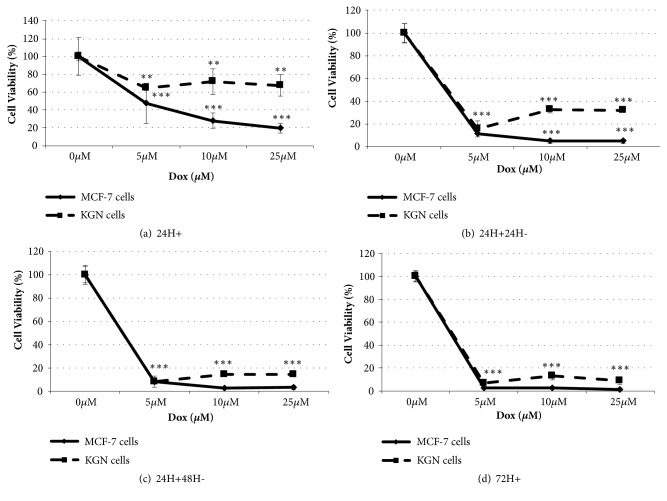
Doxorubicin-induced cytotoxicity. MCF-7 and KGN cells were exposed to Dox 0, 5, 10, 25*μ*M for (a) 24h (24H+), (b) 24h exposure and 24h culture with medium (24H+24H-), (c) 24h exposure and 48h culture with medium (24H+48H-), or (d) 72h continuous exposure (72H+). Complete RPMI (MCF-7) or DMEM/F12 (KGN) without Dox (0*μ*M) was used as a control. Cell viability was assessed by a crystal violet assay, in which cell number was obtained by comparison with a standard curve and % cell viability was calculated from medium control. Means ± SD of 3 independent experiments shown. Data analysed by one-way ANOVA with Tukey's post hoc test. *∗*p ≤ 0.05; *∗∗* p ≤ 0.01, *∗∗∗* p ≤ 0.0001 compared to control.

Doxorubicin killed both MCF-7 and KGN cells (Figure 1). A 24h exposure to 5*μ*M Dox significantly decreased MCF-7 to 46±22% (p<0.0001) and KGN to 65±3% (p<0.01) percent of control (n=3, Figure 1(a)). Cells were exposed to Dox for 24h, then the cells were washed and cultured for an additional 24 or 48h in medium alone (Figures 1(b) and 1(c)) with media replenished at 24h intervals. There was a time-dependent decrease in the numbers of viable cells during the subsequent 48h culture (Figures 1(b) and 1(c)). There were similar numbers of viable cells after 72h continuous exposure to Dox (with media replenishment every 24h, Figure 1(d)) as those after 24h exposure and a further 48h culture (Figure 1(c)).

**Figure 2:**
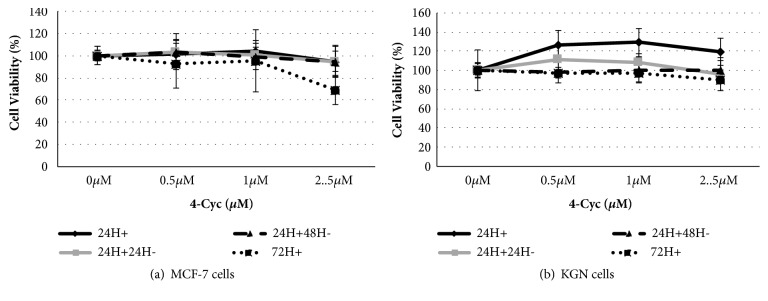
Effect of 4-Cyc on cell viability. (a) MCF-7 and (b) KGN cells were exposed to 4-Cyc 0, 0.5, 1, 2.5*μ*M for 24h exposure (24H+), 24h exposure and 24h culture with media (24H+24H-), 24h exposure and 48h culture with media (24H+48H-), or 72h continuous exposure (72H+). Complete RPMI or DMEM/F12 without 4-Cyc (0*μ*M) was used as a control. Cell viability was assessed by a crystal violet assay, in which cell number was obtained by comparison with a standard curve and % cell viability was calculated from medium control. Means ± SD of 3 independent experiments shown. Data analysed by one-way ANOVA with Tukey's post hoc test.

4-Cyc had no effect on KGN cell viability (Figure 2(a)) and only the longest 72h exposure to the highest concentration (2.5*μ*M) of 4-Cyc significantly reduced the numbers of viable MCF-7 cells to 56354±1657 cells per well (p<0.05).

**Figure 3:**
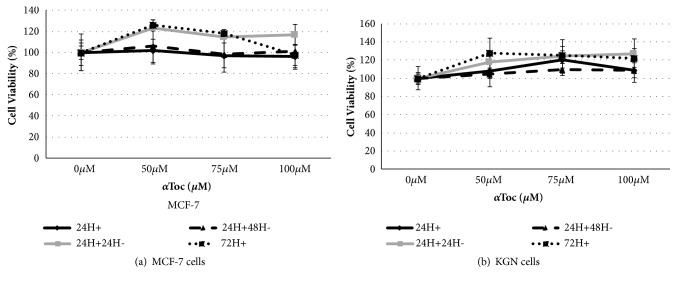
Effect of *α*Toc on cell viability. MCF-7 and KGN cells were exposed to *α*Toc 0, 50, 75, 100*μ*M for 24h exposure (24H+), 24h exposure and 24h culture with media (24H+24H-), 24h exposure and 48h culture with media (24H+48H-), or 72h continuous exposure (72H+). Culture media containing 0.8% DMSO was used as a control. Cell viability was assessed by a crystal violet assay, in which cell number was obtained by comparison with a standard curve and % cell viability was calculated from vehicle control. Means ± SD of 3 independent experiments shown. Data analysed by one-way ANOVA with Tukey's post hoc test.

**Figure 4:**
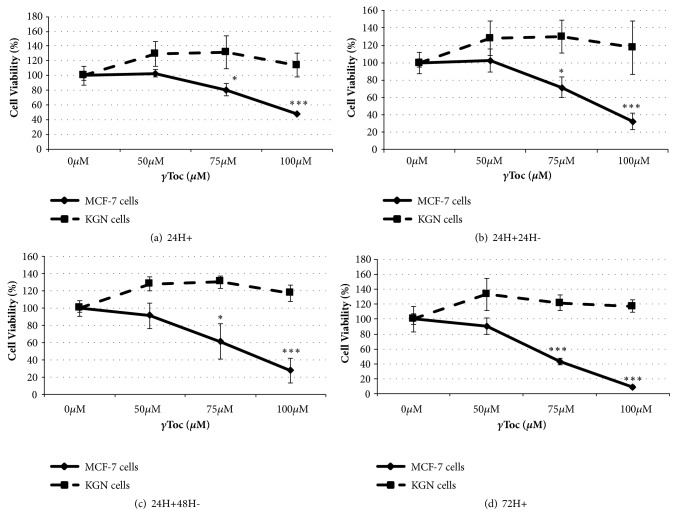
Effect of *γ*Toc on cell viability. MCF-7 and KGN cells were exposed to *γ*Toc 0, 50, 75, 100*μ*M for 24h exposure (24H+), 24h exposure and 24h culture with media (24H+24H-), 24h exposure and 48h culture with media (24H+48H-), or 72h continuous exposure (72H+). 0.8% DMSO in RPMI or DMEM/F12 was used as a control. Cell viability was assessed by a crystal violet assay, in which cell number was obtained by comparison with a standard curve and % cell viability was calculated from vehicle control. Means ± SD of 3 independent experiments shown. Data analysed by one-way ANOVA with Tukey's post hoc test. *∗*p ≤ 0.05; *∗∗* p ≤ 0.01, *∗∗∗* p ≤ 0.0001 compared to control.

Exposure to *α*Toc had no significant effect on MCF-7 or KGN cell viability (Figure 3) but *γ*-Toc was significantly more cytotoxic to MCF-7 cells than to KGN cells (Figure 4). A dose- and time-dependent decrease in MCF-7 cell viability were observed after a 24h or a 72h continuous exposure to *γ*Toc (Figure 4), but increasing concentrations of *γ*Toc had no significant effects on KGN cell viability compared to the vehicle control (Figure 4). The percentage of viable KGN cells after 24h exposure to 100*μ*M *γ*Toc was 113±16% per cells/well, similar to the percentage of viable cells after exposure to the same concentration of *α*Toc (109±13% cells/well, Figure 3).

**Figure 5:**
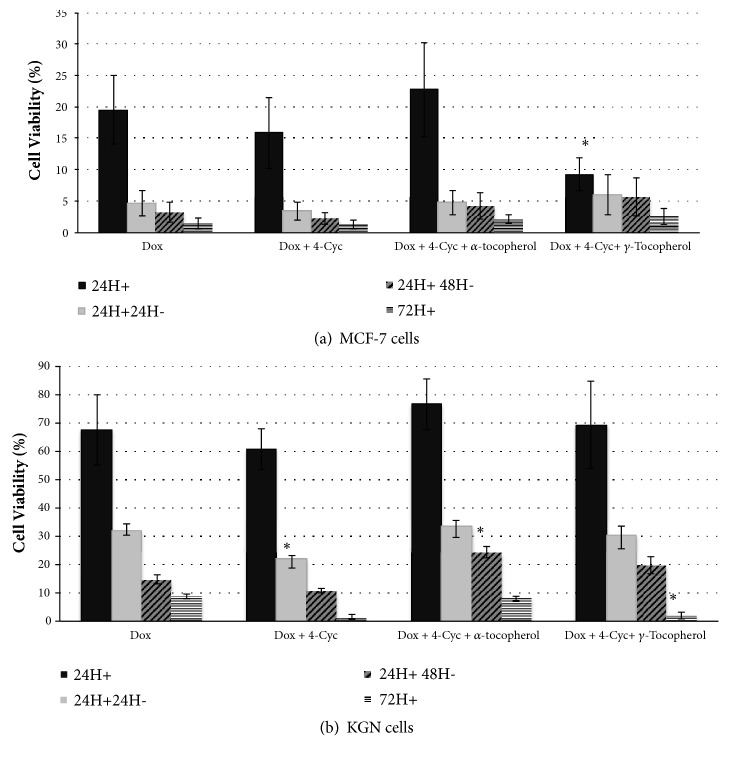
Cytotoxicity of combined chemotherapeutic regime. (a) MCF-7 and (b) KGN cells were exposed to a combination of chemotherapeutics (25*μ*M Dox + 2.5*μ*M 4-Cyc), or a combination of chemotherapeutics + 75*μ*M *α*Toc, or a combination of chemotherapeutics + 75*μ*M *γ*Toc for 24h (24H+); 24h exposure and 24h culture with media (24H+24H-); 24h exposure and 48h culture with media (24H+48H-); or 72h continuous exposure (72H+). Cell viability was assessed by a crystal violet assay, in which cell number was obtained by comparison with a standard cue and % cell viability was calculated from vehicle control. Means ± SD of 3 independent experiments shown. Data analysed by one-way ANOVA with Tukey's post hoc test. *∗*p ≤ 0.05; *∗∗* p ≤ 0.01, *∗∗∗* p ≤ 0.0001 compared to control same concentration of doxorubicin alone (25*μ*M).

The viability of MCF-7 cells was reduced to 31±7% percent of control by a 24h exposure to the low concentration combination of Dox (10*μ*M) and 4-Cyc (1*μ*M), similar to that observed with the same (10*μ*M) concentration of Dox alone (data not shown). When the MCF-7 cells were exposed to the combination of higher concentrations of Dox (25*μ*M) and 4-Cyc (2.5*μ*M) for 24h, the combination also had the same effect as Dox (25*μ*M) alone; viable MCF-7 cells were reduced to 16±6% of control (Figure 5(a)). Adding *α*Toc to this combination had no effect on cell viability (23±7% of control), but the addition of *γ*Toc (75*μ*M) to the combination decreased MCF-7 cell viability to 9±3% cells per well after 24h exposure, significantly lower than Dox alone (p<0.05, Figure 5(a)) or 4-Cyc alone (2.5*μ*M, Figure 2(a), 95±13% of control), or compared to the combination of Dox and 4-Cyc (Figure 5(a)).

The combination of Dox (25*μ*M) and 4-Cyc (2.5*μ*M) caused significantly more KGN cell death than Dox alone (Figure 5(b)). After 72h exposure to this combination there were 1763±1494 KGN cells per well (1.4±1 % of control, Figure 5(b)), significantly lower than those after a 72h exposure to Dox alone (10555±4797, p<0.01), equivalent to 8.7±3.4 percent of control (Figure 5(b)). The addition of *α*Toc to this combination reduced KGN cell death so that it was the similar to Dox alone, 7305±1823 cells per well, equivalent to 7.9±1 percent of control (Figure 5(b)). The addition of *γ*Toc to the combination did not augment the cytotoxicity of Dox and 4-Cyc in KGN cells (Figure 5(a)). Overall, *γ*Toc combined with Dox and 4-Cyc was more cytotoxic towards MCF-7 than KGN cells in the first 24h of culture (Figure 5).

**Figure 6:**
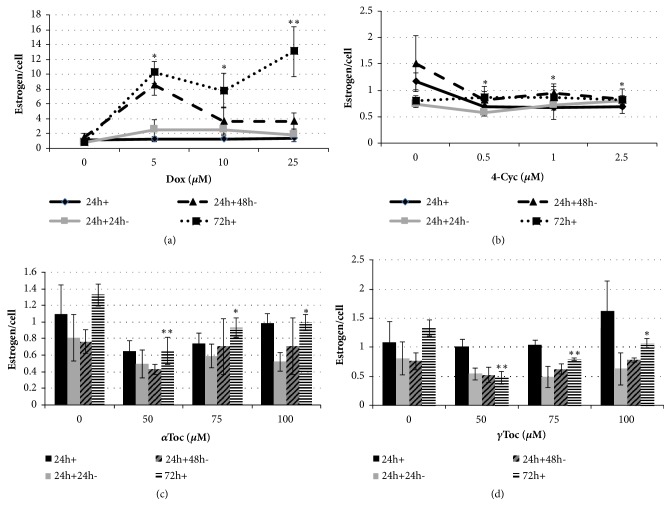
Effect of chemotherapeutics and tocopherols on estrogen production. KGN cells were exposed to Dox (0, 5, 10, 25*μ*M), 4-Cyc (0, 0.5, 1, 2.5*μ*M), *α*Toc (0, 50, 75, 100*μ*M) or *γ*Toc (0, 50, 75, 100*μ*M) for 24h exposure (24h+), 24h exposure and 24h culture with fresh DMEM/F-12 complete medium (24h+24h-), 24h exposure and 48h culture with DMEM/F-12 complete medium (24h+48h-), or 72h continuous exposure where reagents in medium + 10% FCS were replenished every 24h (72h+). Estrogen production was assessed in supernatant at the end of each exposure using an estradiol Enzyme-Linked Immunoassay, in which concentration of estrogen (pg/mL) was obtained by comparison with a standard curve, and estrogen/cell concentration was calculated by dividing pg/mL of estrogen by the number of viable cells in the same well. Means ± SD of 3 independent experiments shown. Data analysed by one-way ANOVA with Tukey's post hoc test. *∗*p ≤ 0.05; *∗∗* p ≤ 0.01, *∗∗∗* p ≤ 0.0001 compared to the same exposure control.

After 24h culture KGN cells produced 1.2±0.1 pg/cell of estrogen and 0.8±0.08 pg/cell in the last 24h of a 72h culture under control conditions (Figures 6(a) and 6(b)). A 24h exposure to 5*μ*M Dox significantly reduced KGN cell viability (Figure 1(a)) but had no effect on estrogen per cell production, which was 1.2±0.03 pg/cell (Figure 6(a)). However, a continuous 72h exposure to Dox, during which media were replenished every 24h and the number of viable cells decreased (Figure 1(d)), caused a significant increase to 13±3 pg/cell (p<0.01, Figure 6(a)) in the last 24h culture period. The same 72h continuous exposure to 2.5*μ*M 4-Cyc had no effect on cell viability (Figure 2(b)) and no effect on estrogen production, which was 0.81±0.08 pg/cell in the last 24h culture period (Figure 6(b)).

When KGN cells were exposed to tocopherols, the 24h+48h^−^ control KGN cells were exposed to almost the same conditions as the 72h+ control cells, 72h* in vitro* with media replenished every 24h. The only difference was that the 72h+ continuously exposed cells were cultured with 0.8% DMSO throughout, whereas the 24h+48h^−^ control KGN cells were only cultured in the presence of 0.8% DMSO for the first 24h. The 72h+ exposure to 0.8% DMSO did not significantly affect KGN cell viability (Figure 3(b)), but it stimulated significantly more estrogen production (1.32±0.07 pg/cell) in the last 24h period of culture than the 24h+48h- exposure which supported production of 0.76±0.14 pg/cell (p< 0.05, Figures 6(c) and 6(d)).

KGN cells in the 0.8% DMSO control produced 1.1±0.4 pg/cell after 24h* in vitro*. The same 24h exposure to *α*Toc had no effect on estrogen per cell production (Figure 6(c)) whereas 100*μ*M *γ*Toc stimulated the production of 1.6±0.5 pg/cell (Figure 6(d)). A 72h continuous exposure to either *α*Toc or *γ*Toc significantly reduced estrogen per cell production compared to control medium containing 0.8% DMSO (Figures 6(c) and 6(d)). The highest (100*μ*M) concentration of *α*Toc and *γ*Toc supported higher levels of estrogen synthesis than the lowest (50*μ*M) concentrations of the tocopherols.

**Figure 7:**
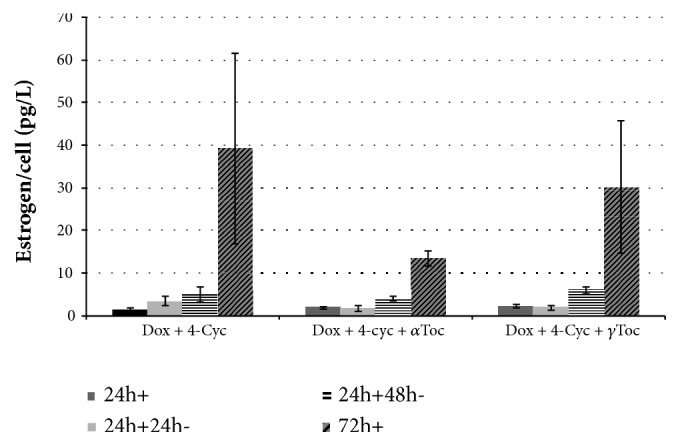
Effect of chemotherapeutics and tocopherols on estrogen production. Combined chemotherapeutic regime (25*μ*M Dox with 2.5*μ*M 4-Cyc), combined regime + 75*μ*M *α*Toc, or combined regime + 75*μ*M *γ*Toc for 24h exposure (24h+), 24h exposure and 24h culture with fresh DMEM/F-12 complete medium (24h+24h-), 24h exposure and 48h culture with DMEM/F-12 complete medium (24h+48h-), or 72h continuous exposure where reagents in medium + 10% FCS were replenished every 24h (72h+). Estrogen production was assessed in supernatant at the end of each exposure by using an estradiol ELISA, in which concentration of estrogen (pg/mL) was obtained by comparison with a standard curve, and estrogen/cell concentration was calculated by dividing pg/mL of estrogen by the number of viable cells in the same well. Means ± SD of 3 independent experiments shown. Data analysed by one-way ANOVA with Tukey's post hoc test. *∗*p ≤ 0.05; *∗∗* p ≤ 0.01, *∗∗∗* p ≤ 0.0001 compared to control same concentration of doxorubicin alone (10*μ*M).

A continuous 72h exposure to the combination of Dox and 4-Cyc reduced cell viability (Figure 5(b)) but stimulated the highest recorded estrogen per cell production; 39±22 pg/cell in the last 24h culture period (Figure 7). This was also higher than the estrogen per cell concentration caused by 72h exposure to Dox alone (Figure 6(a)). The addition of *α*Toc or *γ*Toc to the combination of Dox and 4-Cyc had no statistically significant effect on estrogen per cell production (Figure 7), although it was noted that 72h exposure to the combination of Dox and 4-Cyc with 75*μ*M *α*Toc resulted in 13±2 pg/cell.

## 5. Discussion

The combination of Dox and cyclophosphamide has been used as a standard chemotherapy option for breast cancer patients since 1975 [3, 66]. Although it is a successful treatment for breast cancer [2], it causes premature ovarian failure and infertility [10]. This study showed for the first time that the combination of Dox and 4-Cyc caused the same cytotoxicity to MCF-7 breast cancer cells* in vitro *as Dox alone, but there were different cytotoxic effects towards the KGN ovarian granulosa cell line; the Dox and 4-Cyc combination was significantly more cytotoxic than Dox alone. Similarly, *γ*Toc affected the two cell lines differently; it augmented the cytotoxicity of the Dox and 4-Cyc combination towards MCF-7 cells but did not affect cytotoxicity of the combination towards the KGN cells.

Breast cancer patients are administered multiple cycles of Dox and cyclophosphamide [3], and although this can result in 90% survival for 5y [2], chemotherapeutic-resistant cells are known to cause recurrence of the cancer. The exposure and culture schedules used in this* in vitro* study resulted in only 54% of MCF-7 and 35% of KGN cells being killed in the first 24h of exposure. In our* in vitro* model ‘viable' meant cells were adherent to the floor of the culture vessel, whereas nonadherent dead cells were washed away. Cells with damaged DNA may still function and adhere to the culture vessel, and it is likely that DNA damage is only manifested as cell death or loss in the crystal violet assay when the cell attempts to go through mitosis. Since the doubling time for MCF-7 is 29h [67] and was originally reported as being 46h for the KGN cell line [64], we expected to see further cell loss in the 48–72h following removal of the chemotherapeutics, and this proved to be the case; fewer than 10% of the cells were viable after 72h* in vitro*. We conclude that additional time in culture, sufficient for the MCF-7 to undergo mitosis, would be needed to be able to determine if this surviving ≤10% would give rise to Dox-resistant cells or if these would also die. Further development is required to determine if this* in vitro* system can be used to derive chemoresistant cells.

Resistance or sensitivity to chemotherapeutics* in vivo* is affected by a number of interacting factors including the hepatic clearance of the chemotherapeutics and intracellular levels of metabolising enzymes such as glutathione S-transferase [68] or aldehyde dehydrogenase, which* in vitro* metabolises 4-Cyc to its inactive form [18]. KGN cells were more sensitive to Dox but less sensitive to 4-Cyc than MCF-7 cells. We concluded this because a 72h continuous exposure to 4-Cyc reduced the number of viable MCF-7 cells but had no effect on KGN cells. It is possible that KGN cells express higher levels of aldehyde dehydrogenase than MCF-7 cells and hence metabolised 4-Cyc to its inactive form [62].

A relatively short 24h* in vitro *exposure to 2.5*μ*M 4-Cyc had no effect on MCF-7 cells, although this concentration is two orders of magnitude higher than the plasma concentration (0.02*μ*M) of the pharmacologically equivalent 4-hydroxycyclophosphamide 2-24h after administration of cyclophosphamide* in vivo*. The pharmacokinetics of cyclophosphamide has been well characterised [69–71], but much less is known about the kinetics of the metabolites of cyclophosphamide. The hepatic metabolite 4-hydroxycyclophosphamide has a plasma half-life of only a few minutes* in vivo* [71] because it undergoes spontaneous alteration into phosphoramide mustard [17, 18]. However, phosphoramide mustard may be ionised at physiological pH with a consequent reduction in cytotoxicity, and the oxidation of 4-hydroxycyclophosphamide can produce inactive metabolites [71]. Therefore, the clinically relevant dose of cyclophosphamide necessary to treat breast cancer patients might differ from the* in vitro* effective concentration.

Dox was more cytotoxic to MCF-7 cells than 4-Cyc. Although 2.5*μ*M 4-Cyc did kill MCF-7 cells after 72h continuous exposure, when the same 2.5*μ*M concentration of 4-Cyc was combined with Dox for 72h, the numbers of surviving cells were comparable to those recorded after exposure to Dox alone, suggesting that in this* in vitro *model 4-Cyc did not potentiate the* in vitro* effect of Dox in the MCF-7 cells. Corbett et al. [26] found that the growth of murine mammary adenocarcinomas* in vivo* was slower after administration of Dox as a single agent than after cyclophosphamide alone, meaning that the Dox was more cytotoxic than cyclophosphamide* in vivo*. However, the combination of Dox and cyclophosphamide delayed the* in vivo* development of mammary adenocarcinomas for longer than after the administration of each single agent [26] which suggested therapeutic synergism between the two chemotherapeutics* in vivo*.

The combination of Dox and 4-Cyc reduced MCF-7 viability by 85% whereas exposure to 75*μ*M *γ*Toc for 24h caused a 20% reduction in viable cell numbers. The addition of 75*μ*M *γ*Toc to Dox and 4-Cyc for 24h reduced cell viability by 91%, less than the amount of cytotoxicity predicted by adding the activity of *γ*Toc to Dox and 4-Cyc. More studies using lower concentrations of reagents are needed to determine if there are synergistic interactions between *γ*Toc, Dox, and 4-Cyc.

A long 72h continuous exposure to 2.5*μ*M 4-Cyc had no effect on KGN cell viability nor estrogen per cell production, a 72h exposure to Dox was cytotoxic, and exposure to the combination of Dox and 2.5*μ*M 4-Cyc was more cytotoxic than exposure to Dox alone. This result suggested synergism between Dox and 4-Cyc, but a mechanism for that synergism cannot be deduced from this study. It is possible that 4-Cyc caused DNA crosslinking [18], but this damage was repaired in KGN cells exposed to 4-Cyc alone, whereas the addition of Dox to 4-Cyc prevented the damage from being repaired [27] and hence caused KGN cell death.

In a previous study, KGN cells incubated with androstenedione for 72h synthesised and secreted significant amounts of estrogen into the culture medium [64]. In the present study, a 24h culture in DMEM/F-12 medium containing 10% FCS and ITS resulted in the production of 1.2±0.1 pg/cell, and that rate of production was maintained for 72h when the culture medium was replenished every 24h. Foetal calf serum is rich in fatty acids and cholesterol, the substrate for the whole steroidogenic pathway [72]. Fatty acids, like arachidonic acid, play an essential role in StAR protein expression [73] and the* in vitro* synthesis of steroid hormones such as progesterone and estrogen. In this study, the use of DMEM/F12 with 10% FCS and ITS was enough to support steroidogenesis; androstenedione was not required to support estrogen synthesis and secretion.

Bak et al. [53] reported that estrogen induced the expression of cyclin D1 and c-myc and hence increased mitosis in MCF-7 cells* in vitro*, and that *γ*Toc, but not *α*Toc, inhibited expression of these cell-cycle genes and reduced estrogen-stimulated MCF-7 cell proliferation. The MCF-7 cells in our study were not exposed to estrogen; therefore this was not the cause of the significant cell death caused by *γ*Toc in our study, suggesting that *γ*Toc is cytotoxic through another estrogen-independent mechanism of action. Lee et al. [61] showed that *γ*Toc was cytotoxic to breast cancer cells because it enhanced the transactivation of PPAR*γ* which caused apoptosis and inhibited cell-cycle progression. *γ*Toc has also shown anticancer activity in numerous cancer models, including colon [74], prostate [75], and lung cancer [76] in the absence of estrogen. KGN cells synthesised estrogen, which raises the possibility that there may have been interactions between estrogen and *γ*Toc, but *γ*Toc alone did not cause cytotoxicity towards KGN cells in the presence of 75 to 183 pg/mL estrogen, and neither did *γ*Toc increase the cytotoxicity of the combination of Dox and 4-Cyc, which suggests that the proapoptotic effect that Bak et al. [53] reported in estrogen-stimulated MCF-7 exposed to *γ*Toc does not apply to KGN cells.

Exposure to Dox for 72h caused significant KGN cell death and, counterintuitively, also caused a significant increase in estrogen production per KGN cell. This effect has been reported in other steroid hormone-synthesising reproductive cell lines* in vitro*. An extract from a marine snail was significantly cytotoxic to a human Jar choriocarcinoma placental cell line. As the number of viable cells decreased, secreted progesterone increased [77]. Gross et al. [78] also described dying primary-derived granulosa cells increasing progesterone production. It is possible that the cytotoxic mechanisms of action in these cases disrupted membranes and dysregulated steroidogenesis, resulting in massive overproduction of steroid hormones. This confounding effect might be avoided in future by measuring production of another nonsteroid hormone, AMH, which is important for fertility.

Four test reagents (*γ*Toc, *α*Toc, Dox, and 4-Cyc) were each tested at several different concentrations in four exposure schedules. This generated a relatively high number of test conditions which justified the use of human cell lines. Further studies examining ROS generation and cell death will support the selection of a reduced number of test conditions. At this point MCF-7 cells could be replaced with heterogeneous populations of primary-derived breast cancer cells from different tumour types, and the KGNS could be replaced with 3D primary-derived ovarian follicle culture [79] to better model the effects of chemotherapeutics with or without tocopherols on breast cancer and the ovary.

In summary, 4-Cyc was active because a 72h continuous exposure killed MCF-7 cells and reduced KGN estrogen per cell production. Both *γ*Toc and Dox (applied as single agents) significantly reduced the numbers of viable MCF-7 and KGN cells within 24h of exposure, whilst *α*Toc reduced the cytotoxic effects of the Dox and 4-Cyc combination in KGN cells. The 4-Cyc concentration, despite two orders of magnitude higher than effective clinical plasma concentrations, may have been too low for this* in vitro* model; hence we do not exclude the possibility of therapeutic synergism of the Dox and 4-Cyc combination in MCF-7 cells too. Our hypotheses were partially supported: although the Dox and 4-Cyc combination was not more cytotoxic than Dox alone towards MCF-7 cells, the combination displayed therapeutic synergism towards the ovarian KGN granulosa cells. *γ*Toc, but not *α*Toc, augmented the cytotoxic activity of Dox and 4-Cyc in the MCF-7 cells, but not the KGN cells. This study supports further work to explore the potential of *γ*Toc to increase the chemotherapeutic efficacy of Dox and 4-Cyc against breast cancer cells* in vitro*.

## Figures and Tables

**(a):** 

**Single agents**	**Concentrations (*μ*M)**
Dox	0.5, 10, 25
4-Cyc	0, 0.5, 1, 2.5
*α*Toc	0, 50, 75, 100
*γ*Toc	0, 50, 75, 100

**(b):** 

**Combined agents**		**Concentrations (*μ*M)**
Dox + 4-Cyc	Low	10 (Dox) + 1 (4-Cyc)
Dox + 4-Cyc	High	25 (Dox) + 2.5 (4-Cyc)

Dox + 4-Cyc + *α*Toc	Low	10 (Dox) + 1 (4-Cyc) + 75 (*α*Toc)
Dox + 4-Cyc + *α*Toc	High	25 (Dox) + 2.5 (4-Cyc) 75 (*α*Toc)

Dox + 4-Cyc + *γ*Toc	Low	10 (Dox) + 1 (4-Cyc) + 75 (*γ*Toc)
Dox + 4-Cyc + *γ*Toc	High	25 (Dox) + 2.5 (4-Cyc)+ 75 (*γ*Toc)

## Data Availability

The raw data for cell viability assay and ELISA, used to support the findings of this study, may be released upon reasonable request to the corresponding author, who can be contacted at daniela.figueroa@flinders.edu.au. The graphs used to support the findings of this study are included within the article.

## Abbreviations

Dox: Doxorubicin
4-Cyc: 4-Hydroperoxycyclophosphamide
ROS: Reactive oxygen species
AMH: Anti-Müllerian hormone
*α*Toc: Alpha tocopherol
*γ*Toc: Gamma tocopherol.
